# Clinicopathological analysis of colorectal carcinoid tumors and patient outcomes

**DOI:** 10.1186/1477-7819-12-366

**Published:** 2014-11-30

**Authors:** Hung-Hsin Lin, Jen-Kou Lin, Jeng-Kai Jiang, Chun-Chi Lin, Yuan-Tzu Lan, Shung-Haur Yang, Huann-Sheng Wang, Wei-Shone Chen, Tzu-Chen Lin, Wen-Yih Liang, Shih-Ching Chang

**Affiliations:** Division of Colon and Rectal Surgery, Department of Surgery, Taipei Veterans General Hospital, No 201, Sec. 2, Shih-Pai Road, Taipei, 11217 Taiwan; National Yang-Ming University, No.155, Sec.2, Linong Street, Taipei, 11221 Taiwan; Department of Pathology, Taipei Veterans General Hospital, No 201, Sec. 2, Shih-Pai Road, Taipei, 11217 Taiwan

**Keywords:** Colorectal, Carcinoid tumor, Lymph node metastasis, Clinicopathological

## Abstract

**Background:**

Colorectal carcinoid tumors are often described as being low-grade malignant. The objective of the current study was to address the clinicopathological features and outcomes of patients with colorectal carcinoid tumors.

**Methods:**

A total of 63 patients with colorectal carcinoid tumors were identified and evaluated using surgical pathology files and medical records between January 2000 and June 2012 at the Veterans General Hospital, Taipei, Taiwan.

**Results:**

The median age of the 63 patients was 57.0 years; 38 (60.3%) were male and 25 (39.7%) female. The rectum was the most common tumor site (90.5%). Tumor size was 10.8 ± 7.4 mm, ranging from 2 to 50 mm in diameter. There were 40 patients (63.5%) who received endoscopic treatment for a tumor size of 7.7 ± 4.0 mm, 15 (23.8%) who underwent transanal excision for a mean size of 9.2 ± 4.5 mm and eight (12.7%) who underwent radical surgical resection (mean size: 29.5 ± 13.0 mm). Lymph node metastasis was significantly associated with tumor size. Totally distant metastases (liver) were demonstrated in four (6.3%), patients with mean tumor size of 31.3 ± 9.4 mm (20 to 50 mm). The extent of the disease was associated with survival and the five-year overall survival rate was 92.1%.

**Conclusions:**

With widespread colorectal cancer screening, heightened awareness and improved diagnostic modalities, the incidence of colorectal carcinoid tumors will continue to increase. We demonstrated that small-sized colorectal carcinoid tumors and those localized in the mucosa or submucosa may be safely and effectively removed via endoscopic or transanal local excision.

## Background

Carcinoid tumors are rare, comprising approximately 0.49% of all malignancies [1]. These are the most common neuroendocrine tumors in the gastrointestinal system and most incidences occur in the gastrointestinal tract [2], with the rectum and ileum as the most prevalent tumor sites [1, 3]. Gastrointestinal carcinoids are currently referred to as gastroenteropancreatic neuroendocrine tumors (GEP-NETs). Despite several re-classifications of GEP-NETs by the World Health Organization (WHO), the term ‘carcinoid’ is still used as a synonym for ‘well-differentiated NET’ and the term ‘malignant carcinoid’ is used as a synonym for ‘well-differentiated neuroendocrine carcinoma’. Among such tumors, carcinoids of the colon and the rectum are grouped together in the WHO classification and are distinguished from those of the appendix or the ileum. In the WHO classification, colorectal carcinoids are described as ‘low-grade malignant’, even in the presence of metastasis [4]. Furthermore, the WHO classification defines colorectal carcinoids as ‘benign’ if the tumors are localized in the submucosa, measure 20 mm or less and lack vascular invasion [4]. Although most colorectal carcinoids are localized at the time of diagnosis and have low malignant potential, rectal carcinoids measuring less than 1 cm in size still have malignant potential, and the recorded incidence of metastasis for these tumors ranges from 1.7 to 3.4% [5, 6].

Results related to crucial determinants for metastasis in colorectal carcinoids possibly vary in relation to the small number of cases available in each study. Numerous studies have reported various indicators for metastasis, including a tumor size of 10 mm or more or 20 mm or more, invasion to the muscularis propria, older age, male gender, tumor site, histological growth pattern and mitotic rate [7]. As a result, there has been a disagreement on the therapeutic strategy for use in colorectal carcinoids, particularly in relation to whether local excision or radical resection is suitable for an intermediate tumor size between 10 and 20 mm [8].

The purpose of this study is to review and analyze the clinicopathological features of carcinoid tumors of the colon and rectum at a single institution over a 12-year period.

## Methods

### Patients and clinical data

We retrospectively identified patients using our institution’s pathology database and obtained the corresponding medical records. A total of 63 patients with colorectal carcinoids were enrolled at the Taipei Veterans General Hospital between January 2000 and June 2012. The investigation was approved by the local ethics committee.

Clinical data were prospectively recorded in detail and stored in electronic files. The data base included: (1) name, gender, age, family history and major medical problems of each patient; (2) location, size, gross appearance, stage, differentiation and important pathological prognostic features of the tumor and (3) type of operation, complications, recurrence and follow-up conditions. Pathologic staging of the disease was performed according to the American Joint Committee on Cancer (AJCC) Staging Manual, seventh edition, after review of the surgical specimen and investigation of distant metastases [4].

Rectal carcinoids were defined as tumors located within 15 cm of the anal verge, whereas tumors more than 15 cm above the anal verge were regarded as colonic carcinoids. The tumors were classified into three groups according to tumor size: (1) a tumor diameter of less than 10 mm; (2) a tumor diameter between 10 and 20 mm and (3) a tumor diameter of over 20 mm. Tumor sizes were confirmed through pathology reports. In cases where the tumor size was not clearly described in the pathology report, the size was determined according to the colonoscopy report. Treatment methods were classified as endoscopic resection, transanal resection and radical operation.

All patients were followed up on between three and six months after surgery during the first three years, and annually thereafter. Digital examination, chest X-ray, abdominopelvic ultrasound or computed tomography (CT) scan were used. Recurrence was defined as a local or distant disease diagnosed more than three months after the initial surgery, proven by pathological confirmation or progressively increasing size in imaging studies. Survival time was defined as the time elapsed from the date of diagnosis of the carcinoid tumor until death from all causes, or until 30 November 2013, which was the final date of the analysis used in this study.

### Statistical analysis

The statistical endpoint used in our analysis was patient overall survival from the date of treatment. Group distribution for each clinicopathological trait was compared using the two-tailed Fisher’s exact test and the chi-square test. Numerical values were compared using the Student’s *t*-test and data are expressed as mean ± standard deviation (SD). Kaplan-Meier survival curves were constructed and compared using the log-rank test and multivariate analysis was performed using the Cox proportional hazard model. Statistical analyses were performed using the SPSS package (version 16.0 for Windows, SPSS, Chicago, Illinois, United States).

## Results

A total of 63 patients were enrolled in this study and the population consisted of 38 males (60.3%) and 25 females (39.7%). The mean age at tumor resection was 57.0 ± 12.5 years (range: 26 to 87 years) and the male-to-female ratio was 1.52:1. The mean tumor diameter was 10.8 ± 7.4 mm (range: 2 to 50 mm). The clinicopathological features are outlined in Table 1 and include patient age at diagnosis, gender, tumor location, tumor size and treatment (endoscopic resection, local excision and radical operation). The distribution of surgical methods and tumor size of colorectal carcinoid tumors is showed in Table 2. No patient had carcinoid syndrome at diagnosis. At diagnosis, 49 patients (77.8%) were asymptomatic. The most common presenting symptom was bloody stool (six patients, 9.8%). The other main symptoms included diarrhea (three patients, 4.9%), weight loss (two patients, 3.3%), a palpable anal mass (two patients, 3.3%) and constipation (one patient, 1.6%).

**Table 1:** **Distribution of demographic and clinical characteristics of colorectal carcinoid tumors**

	Total (n = 63)	Rectum (n = 57)	Colon (n = 6)	*P*value
Age (mean ± SD, years)	57.0 ± 12.5	57.2 ± 12.6	54.5 ± 10.8	1.000
<65	42 (66.7)	38 (66.7)	4 (66.7)	
≥65	21 (33.3)	19 (33.3)	2 (33.3)	
Gender	1.52: 1			0.738
Male	38 (60.3)	34 (59.6)	4 (66.7)	
Female	25 (39.7)	23 (40.4)	2 (33.3)	
Tumor size (mean ± SD, mm)	10.8 ± 7.4	10.1 ± 6.4	17.2 ± 15.2	0.414
<10	41 (65.1)	37 (65.0)	4 (66.7)	
10 - 20	10 (15.9)	10 (17.5)	0 (0.0)	
≥20	12 (19.0)	10 (17.5)	2 (33.3)	
Treatment				0.149
Endoscopic resection	40 (63.5)	36 (63.2)	4 (66.7)	
Local excision	15 (23.8)	15 (26.3)	0 (0.0)	
Radical operation	8 (12.7)	6 (10.5)	2 (33.3)	

The values in parentheses are presented as percentages.

**Table 2:** **The distribution of surgical methods and tumor size of colorectal carcinoid tumors**

Tumor size (mm)	Surgical methods		
	Endoscopic	Local excision	Radical operation
<10	30	10	1
10 - 20	7	2	1
>20	3	3	6
Total	40	15	8

A total of nine patients were diagnosed as having lymph node metastasis by pathologic investigation or imaging studies. Six patients who underwent low anterior resection (LAR) were identified by histological examination as having lymph node metastasis, and two patients who underwent only endoscopic resection because of unresectable distant metastasis were suspected of having lymph node metastasis on the basis of CT scans. One patient suffered obstruction of the colon and underwent a transverse loop colostomy, but later died from unresectable distant metastasis.

Metastasis to the lymph nodes was significantly associated with tumor size. No lymph node metastasis was observed for tumors smaller than 10 mm, 10% lymph node metastasis was observed for tumors between 10 and 20 mm and 58.3% was observed for tumors larger than 20 mm (Table 3). Distant metastasis at diagnosis was assessed through CT scans or pathological examinations in four cases. All patients with distant metastasis had tumors larger than 20 mm (Table 4). Results of the TNM stage classification according to the AJCC are shown in Table 5.

**Table 3:** **Rate of lymph node metastasis in 63 colorectal carcinoid tumors**

Tumor size (mm)	Total (n = 63)	Rectum (n = 57)	Colon (n = 6)
<10	0/41	0/37	0/4
10 - 20	1/10 (10.0)	1/10 (10.0)	-
>20	7/12 (58.3)	7/10 (70.0)	0/2
Total	8/63 (12.7)	8/57 (14.0)	0/6

The values in parentheses are presented as percentages.

**Table 4:** **Rate of distant metastasis in 63 colorectal carcinoid tumors**

Tumor size (mm)	Total (n = 63)	Rectum (n = 57)	Colon (n = 6)
<10	0/41	0/37	0/4
10 - 20	0/10	0/10	-
>20	4/12 (33.3)	4/10 (40.0)	0/2
Total	4/63 (6.3)	4/57 (7.0)	0/6

The values in parentheses are presented as percentages.

**Table 5:** **TNM staging of colorectal carcinoid tumors according to AJCC**, **seventh edition (n = 63)**

TNM stage (clinical or pathological)		Number (%)
I	T1aN0M0	41 (65.1)
	T1bN0M0	9 (14.3)
IIa	T2N0M0	4 (6.3)
IIb	T3N0M0	1 (1.6)
IIIa	T4N0M0	0
IIIb	Any T, N1M0	4 (6.3)
IV	Any T, any N, M1	4 (6.3)

T1, tumor invades lamina propria or submucosa and size ≤ 2 cm; T1a, tumor size < 1 cm in greatest dimension; T1b, tumor size 1 to 2 cm in greatest dimension; T2, tumor invades muscularis propria or size > 2 cm with invasion of lamina propria or submucosa; T3, tumor invades through the muscularis propria into the subserosa, or into non-peritonealized pericolic or perirectal tissues; T4, tumor invades peritoneum or other organs; N0, no regional lymph node metastasis; N1, regional lymph node metastasis; M0, no distant metastasis; M1, distant metastasis.

**Figure 1:**
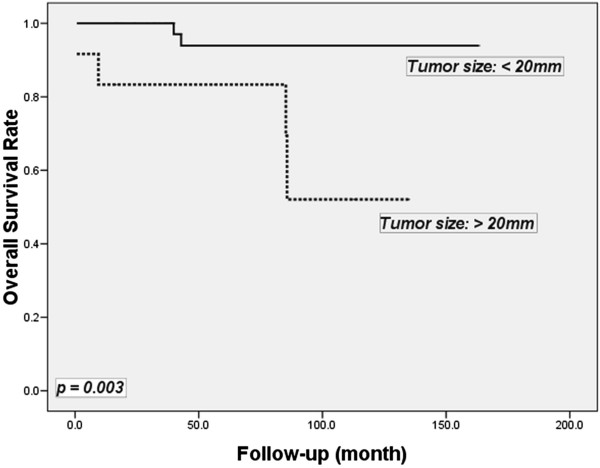
**Kaplan**-**Meier plot illustrating five**-**year overall survival by tumor size.**

The mean length of the follow-up period in this study was 77.1 months. Two patients were lost to follow-up. The five-year overall survival rate was 92.1%. For patients with tumors smaller than 20 mm, the five-year overall survival rate was 94.0%, which was significantly better than those with tumors larger than 20 mm (83.3%; *P* = 0.003) (Figure 1). However, if the cut-off value of the tumor size was defined as 10 mm, the five-year overall survival rates were observed to be similar between patients with tumors larger than, and less than 10 mm (90.9 versus 92.1%; *P* = 0.08).

For the 10 patients with tumors between 10 and 20 mm, seven patients underwent colonoscopic resection, two patients underwent transanal excision, and one patient received radical low anterior resection. During the follow-up period, a 76-year-old male patient developed local recurrence in the rectum and underwent low anterior resection for a 2.0-cm-sized rectal carcinoid. The original pathology revealed invasion of the muscularis propria and two out of 12 lymph nodes were positive for metastasis.

Overall, seven patients in the cohort (11.1%) had a synchronous second primary tumor at the time of carcinoid diagnosis. This included five patients with colorectal adenocarcinoma, one patient with pancreatic adenocarcinoma and one patient with endometrial adenocarcinoma.

## Discussion

In general, the incidence of carcinoid tumors of the colon is reported to be very low and comprises between 4 and 8% of all carcinoid tumors in the digestive system [1, 8, 9]. With widespread colorectal cancer screening, heightened awareness and improved diagnostic modalities, the incidence of early detection of colorectal carcinoid tumors should continue to increase. Although our study was limited to the colon and the rectum, a tumor incidence of 9.5% was observed in the colon, which is slightly higher than the generally accepted incidence rate. The predominance of males over females with rectal carcinoids was observed by Yoon *et al*. [10] (ratio 1:15), Konishi *et al*. [8] (ratio 1:2.2), and Jetmore *et al*. [11] (ratio 1:1.7). In addition, the same predominance was observed in our study (ratio 1:1.52). Carcinoid syndrome was not observed in the patients in our study, which corresponds to prior observations that most colorectal carcinoids are non-functioning and that carcinoid symptomatology is rare (less than 5%) [7].

In the recently revised AJCC cancer staging, carcinoid tumors are classified as a malignant disease. The stage classifications are based on tumor size, the involved layer of the bowel wall and the presence or absence of lymph node or distant metastasis. According to this staging system, our series of cases are classified into 48 stage I cases, four stage IIa cases, one stage IIb case, no IIIa cases, four stage IIIb cases and four stage IV cases. The staging results reflect that most cases were early lesions and were incidentally detected by colonoscopy screening.

A population-based study in Japan reported lymph node metastasis of 3.7% for rectal carcinoids with sizes of 5 mm or less, and 9.7% lymph node metastasis for tumors with sizes of less than 10 mm [9]. These results complicate decisions regarding treatment methods, even in small-sized rectal carcinoids. In our study, only one patient with a rectal carcinoid size between 10 and 20 mm developed regional lymph node metastasis (10.0%). However, if lymph node metastasis is to be accurately predicted, additional studies on the risk factors are required and treatment methods should be determined considering various risk factors [2, 12].

Although tumor size is often cited as the most important prognostic indicator for carcinoid tumors, the best indicators are actually the evidence of invasive growth and the presence of regional or distant metastasis. In our study, no cases of distant metastasis in patients with a tumor size of less than 20 mm were observed. The five-year survival rates for patients with tumor sizes greater than and less than 20 mm, were 83.3% and 94.0%, respectively (*P* = 0.003). Li *et al*. [13] reported that rectal and sigmoid carcinoids measuring more than 2 cm or those that invaded muscle layers (T2) or beyond, or those with distant metastasis had significantly more frequent metastases and a worse survival rate. Patients with these tumors should be treated aggressively and should not be treated using excisional biopsy.

In the case of rectal carcinoids, the risk of lymph node metastasis and distant metastasis varies depending on tumor size. Thus, regarding tumor size is the most important factor in deciding an appropriate treatment method. The reported risk of lymph node metastasis for rectal carcinoids smaller than 10 mm is usually less than 3%; thus, regional treatment including colonoscopic resection is appropriate [3]. However, radical surgery is recommended for tumors larger than 20 mm, where the risk of metastasis is known to be 60 to 80%. Shields *et al*. [14] reported that a tumor size of more than 10 mm and lymphovascular invasion were significantly associated with the presence of nodal disease, rendering mesorectal excision advisable. Treatment methods for tumors with sizes between 10 and 20 mm are controversial and no established treatment guideline exists.

In our present study, there was no lymph node metastasis observed in relation to tumors smaller than 10 mm, 10% in relation to tumors between 10 and 20 mm and 58.3% in relation to tumors larger than 20 mm. Although we are unable to draw specific treatment recommendations based on the results of the current study because of its retrospective nature and small numbers in each subgroup, it appears that rectal carcinoids measuring less than 10 mm can be safely managed by local excision. In addition, local excision is usually recommended for tumors between 10 and 20 mm, but radical surgery should be considered if there is evidence of lymph node metastasis or lymphovascular invasion on biopsy. In contrast, for tumors larger than 20 mm, radical surgery is mandatory. To rule out distant metastasis, imaging studies, such as CT or magnetic resonance imaging, are recommended for patients with rectal carcinoids larger than 20 mm.

In this study, we discovered that seven patients (11.1%) had a synchronous second primary tumor at the time of carcinoid diagnosis. Associated malignancies were frequently identified in conjunction with carcinoids in the rectum and/or sigmoid (18 of 141 patients; 12.8%) [13]. Of all the gastrointestinal tract carcinoids, colorectal carcinoids are reported to have a rate of second primary malignancy in the range of 5 to 32%. The most common site of associated non-carcinoid malignancies was the gastrointestinal tract, which involved between 32 and 62% of all tumors, followed by the genitourinary tract (range: 9 to 22%) and the lung and/or bronchial system (range: 9 to 13%) [11, 13, 15–18]. The etiology for high risk of second primary neoplasms associated with carcinoid tumors remains unclear. Thus, our data and those from other studies strongly suggest that when a carcinoid tumor is identified in a patient, there is a need for close surveillance of the gastrointestinal tract, respiratory system and genitourinary tract, and that long-term follow-up is recommended for patients in order to identify any delayed metastasis or secondary malignancies.

## Conclusions

In this study, we demonstrated that patients with small-sized colorectal carcinoid tumors that are localized in the mucosa or submucosa may be safely and effectively removed via endoscopic or transanal local excision. Patients with colorectal carcinoid tumors generally have a good prognosis, but long-term follow-up is recommended for patients with carcinoid tumors in order to identify any delayed metastasis or secondary malignancies.

## Consent

Written informed consent was obtained from the patient for publication of this report and any accompanying images. A copy of the written consent is available for review by the Editor-in-Chief of this journal.

## Abbreviations

AJCC: American Joint Committee on Cancer
CT: Computed tomography
GEP-NETs: Gastroenteropancreatic neuroendocrine tumors
WHO: World Health Organization.
